# Intimate partner violence among pregnant women attending a low-resource primary care facility in Ghana

**DOI:** 10.1371/journal.pone.0310169

**Published:** 2024-09-09

**Authors:** Stephen Tetteh Engmann, Roberta Lamptey, Henry Jeremy Lawson, Gerhard Ofori-Amankwah

**Affiliations:** 1 Family Medicine Unit, Manna Mission Hospital, Accra, Ghana; 2 Family Health University College, Accra, Ghana; 3 Polyclinic/ Family Medicine Department, Korle Bu Teaching Hospital, Accra, Ghana; 4 Department of Community Health, University of Ghana Medical School, Accra, Ghana; 5 Primary Care & Postgraduate Training Division, Nyaho Medical Centre, Accra, Ghana; Salale University, ETHIOPIA

## Abstract

**Background:**

Intimate partner violence in pregnancy is a significant public health issue that has several detrimental effects. Pregnant women subjected to intimate partner violence (IPV) have a higher risk for adverse pregnancy outcomes.

**Objective:**

The aim of the study was to determine the prevalence, patterns and factors associated with intimate partner violence among pregnant women attending a primary care antenatal clinic.

**Methods:**

A quantitative cross-sectional study design was employed to study intimate partner violence among 269 pregnant women in Accra, Ghana between July and October 2021. Participants were selected by systematic sampling technique. The self-reported Composite Abuse Scale was used to assess and classify intimate partner violence. Socio-demographic, clinical (obstetric) and behavioural characteristics were obtained with a structured questionnaire. Associations were determined between independent and dependent variables using the chi-squared test, and logistic regression with adjusted odds ratio (AOR). The statistical significance level was set at a p-value ≤ 0.05.

**Results:**

The prevalence of IPV was 11.2%. The prevalence of emotional/psychological abuse, harassment/controlling behaviour, physical abuse, sexual abuse and severe combined abuse were 12.3%, 13.0%, 8.2%, 3.3% and 8.9% respectively. Pregnant women who were employed had reduced odds of experiencing IPV [AOR = 0.16 (95%CI: 0.05–0.47), p = 0.001], however, the past experience of violence [AOR = 4.9 (95%CI: 1.06–22.96), p = 0.042], alcohol use by women [AOR = 7.8 (95%CI: 1.63–37.42), p = 0.01], and partners’ alcohol consumption [AOR = 10.0 (95%CI: 3.22–31.26), p<0.001] were associated with increased odds of IPV.

**Conclusions:**

There is a high prevalence of IPV among pregnant women in this study from a resource-limited setting. The factors found to be associated with IPV in pregnancy were the employment status of women, alcohol consumption by women or their partners and a previous history of violence. Healthcare providers in primary care need to recognize IPV as a medical condition that can occur in pregnancy and be ready to assist and manage the victims when it is detected.

## Introduction

Intimate partner violence (IPV), also referred to as intimate partner abuse, is any behaviour by an intimate partner that causes physical, sexual or psychological harm, and includes physical aggression, sexual coercion, psychological abuse and controlling behaviour [1]. A systematic review and meta-analysis of global data reports an estimated overall prevalence of intimate partner violence (IPV) during pregnancy to be 25% [2]. The IPV prevalence in pregnancy in Asia, Europe, North America and South America are reported to be 32.1%, 5.1%, 20.4%, and 25.6% respectively [2]. In Sub-Saharan Africa (SSA), the pooled prevalence of IPV in pregnancy is 34.9% [3] which ranks higher than the global prevalence. In Ghana, data from the Demographic and Health Survey (DHS) shows that 5% of pregnant women experience physical IPV during pregnancy [4]. In a tertiary care facility in Ghana, the prevalence of domestic violence by an intimate partner was reported to be 31.1% during pregnancy [5]. There is a high prevalence of IPV among pregnant women [6–8] which could be due to their vulnerability and dependence, both physically and emotionally, on their partners [6]. Pregnancy indicates a period of heightened vulnerability to IPV due to changes in women’s circumstances [9].

Studies allude to inequalities in IPV across low- and middle-income countries [10, 11]. It has been reported that younger pregnant women from impoverished and less empowered backgrounds are more susceptible to IPV [11]. Pregnant women are more likely to be in intimate relationships than non-pregnant people, which increases their likelihood of experiencing IPV [12]. Furthermore, IPV in pregnancy can be significantly influenced by the nature of the pregnant woman’s relationship with her partner [12]. The existing evidence, however, does not reflect the full burden of the problem of IPV during pregnancy [6].

Intimate partner violence during pregnancy is found to be directly related to adverse pregnancy outcomes and both fatal and non-fatal health consequences in both pregnant women and their offspring [4]. Data from the World Health Organization (WHO) has shown that IPV is linked to a two-fold rise in induced abortions, as well as a 16% increase in low-birth-weight newborns and a 43% increase in pre-term births [13]. It is reported that the risk of low birth weight and preterm birth increases when pregnant women are exposed to multiple forms of IPV [14].

Economic hardships and resource scarcity have been found to create a context for conflict and abuse between partners [15]. Intimate partner violence is strongly correlated with adversity, especially poverty, during the antenatal period [16]. There are sociodemographic and behavioural factors that increase the risk of IPV in pregnant women as well as obstetric factors which include obstetric history and whether the pregnancy was planned or not [17–19]. In many primary care settings in Ghana, there appears to be limited published literature on intimate partner violence among pregnant. An analysis of factors associated with IPV in pregnancy in a resourced-constrained setting like Ghana and in primary care practice, which is the first contact between pregnant women and the healthcare delivery system, provides an opportunity to identify these factors and influence policy measures aimed at addressing IPV which is regarded as a public health concern globally [13].

Primary care providers are well-positioned to identify and address at-risk behaviour for IPV promptly through quality patient-physician interactions built on trust [20]. Through strategies for identifying IPV including screening and case finding in patients with suspected signs or symptoms presenting at the primary care level, management can be instituted [21]. This study, therefore, sought to determine the factors associated with IPV among pregnant women attending a primary care antenatal facility, using a cross-sectional design. The study also sought to determine the prevalence and patterns of IPV among pregnant women at the study site. Furthermore, findings from this research would contribute to the data and body of knowledge on the associated factors of IPV among pregnant women, especially in primary care settings in low-resource countries.

## Materials and methods

### Study design and setting

This was a cross-sectional quantitative hospital-based study carried out from July to October 2021, at the Manna Mission Hospital in the Ledzokuku municipality of the Greater Accra region of Ghana. Manna Mission Hospital is a 40-bed hospital that provides General practice and Family Medicine, general surgery, obstetrics and gynaecology, and paediatric services at the primary and secondary levels of care [22]. Maternal health services at the facility include the provision of antenatal care (ANC) services at the outpatient antenatal clinic. The total antenatal registrants at the facility in 2019 and 2020 were 1,081 and 1,111 respectively. The facility is located in an urban community just east of the capital of Ghana, Accra. There are also several semi-urban communities within the catchment area of the facility in the Municipality. According to the Ghana Statistical Service District Analytical Report, the predominant occupations of the people are trading and marketing, manufacturing, craftsmanship and related industries [23]. Ga ethnicity is the most common ethnic group, followed by Akans [23].

### Sampling and study procedure

Using an estimated population of 723 pregnant women for the proposed period of the study, the sample size determination was calculated with Yamane’s formula [24] as follows:

$$\text{n}=\frac{\text{N}}{1+\text{N}{\left(\text{e}\right)}^{2}}$$

N = Population size and n = Sample size,

e = Level of precision (0.05) with 95% confidence level.

$$\text{n}=\frac{723}{1+723*{\left(0.05\right)}^{2}}$$

n = 258 (approximated to the nearest whole number).

A non-response rate of 10% was added, resulting in a final sample size of 284. A systematic sampling technique was used with a sampling interval (K^th^) of 3 to recruit participants. This was derived by dividing 723 by the number of pregnant women to be selected which was 284.

All pregnant women who were attendants at the antenatal care (ANC) clinic, and who could communicate in either English, Twi, or Ga, were eligible to participate in the study. The data collection instruments were translated into these local languages and administered in the preferred language of the participants. Pregnant women without an identified intimate partner at the time of recruitment, and those requiring hospital admission, were excluded. The aim and procedure for the study were read and explained to eligible participants and written informed consent for those 18 years and above, or assent for participants below 18 years was obtained. Data collection was done within the privacy of the consulting rooms at the ANC clinic using a pretested questionnaire.

### Operational definition of terms and variables

Intimate partner violence: This is represented by individuals who have scores of 7 or more on the Composite Abuse Scale.

Severe Combined Abuse: The eight items that characterize severe physical abuse, as well as the physical isolation aspects of psychological abuse. (2, 5, 7, 15, 18, 22, 25, 26), and 2 items (15 and 25) that represent sexual violence on the Composite Abuse Scale.

Physical Abuse: The 7 items of the less severe physical abuse items on the Composite Abuse Scale (6, 10, 14, 17, 23, 27, 30).

Psychological/Emotional Abuse: The 11 items that include verbal, psychological, dominance and social isolation abuse on the Composite Abuse Scale (1, 4, 8, 9, 12, 19, 20, 21, 24, 28, 29).

Harassment/controlling behaviour: The 4 items that are about actual harassment on the Composite Abuse Scale (3, 11, 13, 16).

Intimate Partner: A husband, as well as informal partnerships, including co-habiting, dating relationships and unmarried sexual relationships.

Trimester: A period of three or about three months of human gestation. The first trimester is 1 to 12 weeks of gestation, the second trimester is 13 to 27 weeks of gestation and the third trimester is defined as the period from 28 weeks of gestation up to delivery.

### Data collection instrument and measures

The dependent variable was “intimate partner violence within the preceding 12 months”. The Composite Abuse Scale (CAS) was used to gather data on IPV. The CAS consists of 30 questions presented in a six-point format, with the options "never," "only once," "several times," "monthly," "weekly," or "daily." Respondents were divided into abused and non-abused groups using a cutoff score of 7 as recommended by Hegarty et al. [25]. The CAS consists of subscales that measure Severe Combined Abuse, Sexual Abuse, Harassment, Emotional Abuse, and Physical Abuse. Validation studies have shown a high internal consistency and reliability with Cronbach’s alphas >0.85 and item-total correlation which is greater than 0.5 [26].

The independent variables, comprising the sociodemographic, obstetrics and behavioural characteristics of the study participants/partners were obtained using a structured questionnaire. The Patient Health Questionnaire-2 (PHQ-2) was used to screen for depression among the participants. The PHQ-2 offers a scoring range from 0–6. Validation studies identified a PHQ -2 cut-off score of 3 to have optimal discriminatory power for the diagnosis of Depression [27].

### Data analysis

Data were entered into Microsoft Excel and cleaned before exporting to STATA 15 for analysis. The various variables were analyzed using frequencies, while proportions or percentages were used to determine the prevalence and patterns of IPV. The Chi-square test was used to determine the association between categorical variables and IPV. Multivariate logistic regression was done to identify factors associated with IPV during pregnancy. The variance inflation factor (VIF) was used to identify multicollinearity between independent variables and the strength of that correlation. Highly correlated independent variables (VIFs > 5) were removed from the model. The variable selection was based on literature and the model selection strategy used for the multiple regression model was the backward elimination method. Adjusted *R*^*2*^ was used to determine the strength of the model fit. The significance level was set at p-value ≤ 0.05.

### Ethical consideration

Ethical approval for this study was obtained from the Ghana Health Service–Ethical Review Committee (GHS-ERC 037/12/20). All participants were duly informed about the purpose of the study. They were encouraged to seek clarity to lingering questions about the study following which written informed consent was obtained prior to enrolment in the study. Parents/guardians of study participants below 18 years were required to sign informed consent forms on behalf of their wards, while these participants were required to provide assent. All participants were informed that they could opt out of the study at any moment without incurring any consequences. To preserve privacy, questionnaires were administered to participants alone in the consulting room and to maintain anonymity, each entry was assigned a code in a codebook. Persons who were experiencing IPV were referred to the appropriate community-based social and psychological professional resources, such as the domestic violence and victims support unit.

## Results

Out of the total sample size, data from 269 consenting participants was used for the analysis resulting in a response rate of 94.7%. The age of participants ranged from 17 to 43 years with a mean age of 29 years (SD±5.08) years. The peak age group was 25 to 34 years, accounting for 65.1% of the participants. The majority (66.5%) of the participants were married. Among those who were not married, 57 (21.2%) were single and 33 (12.3%) were cohabiting. The most prevalent type of marriage was monogamy which constituted 95% of married participants. Most participants (76.9%) were employed, and the most common level of education among them was secondary school, which accounted for 63.6% of the total. The socio-demographic characteristics of all 269 participants recruited for the study are shown in Table 1. The overall prevalence of IPV was 11.2%. The prevalence of emotional abuse, harassment, physical abuse, sexual abuse and severe combined abuse from the composite abuse subscales were 12.3%, 13.0%, 8.2%, 3.3% and 8.9% respectively.

**Table 1:** Socio-demographic characteristics of study participants.

Characteristics	Frequency (N = 269)	Percentage (%)
**Age (years)**–mean (sd), range	29 (5), 17–43	
**Age group of participants (years)**		
15–24	49	18.2
25–34	175	65.1
35–44	45	16.7
**Marital Status**		
Married	179	66.5
Single	57	21.2
Co-habitation	33	12.3
**Length of Relationship (in years)**		
Less than 1 year	35	13.0
1–5	173	64.3
6–10	47	17.5
11 years and above	14	5.2
**Type of Marriage**		
Monogamous	170	95.0
Polygamous	9	5.0
**Highest level of Education**		
Primary	35	13.0
Secondary	171	63.6
Tertiary	57	21.2
No formal education	6	2.2
**Employment Status**		
Employed	207	76.9
Unemployed	62	23.1
**Religion**		
Christianity	256	95.2
Islam	12	4.4
Traditional	1	0.4

Tables 2 and 3 show the obstetric characteristics and behavioural factors of participants respectively. The majority (79.5%) of the participants were multigravida compared to 20.5% of primigravida women. Furthermore, 34.6% had pregnancies that were unplanned at the time of the study. Booking for the antenatal clinic for almost all of the participants was done in either the first or second trimester of the current pregnancy. Concerning pregnancy outcomes of previous pregnancies, 90 (33.5%) had a history of miscarriage or abortion, 15 (5.6%) had a history of stillbirth, and 6 (2.2%) had a history of preterm delivery. The behavioural characteristics of the partners of participants showed that cigarette smoking in the last 12 months and partner’s use of recreational drugs in the last 12 months was 1.5% each. Partner alcohol use in the last 12 months was also 27.5% of all the 269 participants.

**Table 2:** Clinical (Obstetric) characteristics of participants.

Characteristics	Frequency (N = 269)	Percentage (%)
**Gravidity**		
Primigravida	55	20.5
Multigravida	214	79.5
**Parity**		
Nulliparity	80	29.7
Primiparity	94	34.9
Multiparity	93	34.6
Grand multiparity	2	0.7
**Current Pregnancy Unplanned**		
No	176	65.4
Yes	93	34.6
**Gestational Age of Pregnancy**		
First trimester (1 to 12 weeks)	28	10.4
Second trimester (13 to 27 weeks)	132	49.1
Third trimester (28weeks to delivery)	109	40.5
**Gestational age at booking**		
First trimester (1 to 12 weeks)	130	48.3
Second trimester (13 to 27 weeks)	130	48.3
Third trimester (28weeks to delivery)	9	3.4
**History of miscarriage or abortion**		
No	179	66.5
Yes	90	33.5
**History of stillbirth**		
No	254	94.4
Yes	15	5.6
**History of preterm delivery**		
No	263	97.8
Yes	6	2.2

**Table 3:** Behavioural characteristics of study participants.

Characteristics	Frequency (N = 269)	Percentage (%)
**Participant’s experience of conflict or dissatisfaction in the relationship**		
No	227	84.4
Yes	42	15.6
**Participant’s past experience of violence committed by parents or another family member**		
No	246	91.4
Yes	23	8.6
**Participant’s alcohol use in the last 12 months**		
No	254	94.4
Yes	15	5.6
**Participant cigarette smoking in the last 12 months**		
No	269	100
Yes	0	0
**Participant use of recreational drugs**		
No	269	100
Yes	0	0
**PHQ 2 Screening for Depression**		
Not Depressed (PHQ < 3)	242	90.0
Depressed (PHQ ≥ 3)	27	10.0

The sociodemographic factors associated with IPV in bivariate analysis were participants’ employment status (χ2 = 36.221, p = 0.000), employment status of their partners (χ2 = 7.291, p = 0.007), and partner’s highest level of education (χ2 = 13.038, p = 0.005). The obstetric or clinical factors associated with IPV in bivariate analysis were unplanned pregnancy (χ2 = 63.901, p = 0.000), the gestational age at which the participants presented for booking (χ2 = 11.183, p = 0.004), and a history of stillbirth (χ2 = 3.859, p = 0.049). The behavioural factors associated with IPV on bivariate analysis were participants’ experience of conflict or dissatisfaction in the relationship (χ2 = 154.799, p = 0.000), participant’s past experience of violence committed by parents or another family member (χ2 = 26.524, p = 0.000), alcohol consumption by participants (χ2 = 52.766, p = 0.000) and partner alcohol consumption (χ2 = 52.766, p = 0.000). Among the participants, there was a statistically significant association between a positive screen for depression and IPV. (χ2 = 106.217, p = 0.000). The chi-square test is provided in the S1 Table (Supporting information).

Multiple logistic regression analysis in Table 4 shows the factors associated with IPV identified among the participants. The adjusted regression analysis demonstrated that pregnant women who were employed had reduced odds (84%) of experiencing IPV compared to unemployed pregnant women. [AOR = 0.16 (95% CI = 0.05–0.47), p<0.01]. For women who have experienced violence committed by parents or family members in the past, the odds of experiencing IPV is 4.9 times as great as the odds of pregnant women without a previous experience of violence [AOR = 4.9 (95% CI = 1.06–22.96), p<0.05]. Pregnant women who used alcohol in the last 12 months had 7.8 times the odds of experiencing IPV compared to pregnant women who didn’t take alcohol. [AOR = 7.8 (95% CI = 1.63–37.42), p = 0.01]. Furthermore, women whose partners used alcohol in the last 12 months had 10 times the odds of experiencing IPV compared to women whose partners didn’t use alcohol [AOR = 10.0 (95% CI = 3.22–31.26), p<0.001].

**Table 4:** Factors associated with intimate partner violence among pregnant women.

Variables	Coef. β	aOR	95% CI	p-value
**Marital status**				
Single	0	ref		
Married	-0.049	0.952	0.234–3.873	0.945
Cohabitation	-0.916	0.400	0.053–3.033	0.376
**Employment Status of Participants**				
Unemployed	0	ref		
Employed	-1.864	0.155	0.051–0.468	0.001*
**Parity**				
Nulliparity	0	ref		
Primiparity	-0.117	0.889	0.208–3.805	0.874
Multiparity	0.155	1.168	0.268–5.094	0.837
**History of miscarriage**				
No	0	ref		
Yes	0.345	1.412	0.459–4.338	0.547
**History of stillbirth**				
No	0	ref		
Yes	0.411	1.509	0.156–14.574	0.722
**History of prematurity**				
No	0	ref		
Yes	0.685	1.984	0.142–27.659	0.610
**Participant’s past experience of violence**				
No	0	ref		
Yes	1.597	4.938	1.062–22.962	0.042*
**Participant alcohol use**				
No	0	ref		
Yes	2.056	7.817	1.633–37.415	0.010*
**Partner alcohol use**				
No	0	ref		
Yes	2.306	10.032	3.219–31.261	0.000*
**Partner use of recreational drugs**				
No	0	ref		
Yes	1.081	2.949	0.061–141.547	0.584

(* statistically significant), aOR-adjusted odds ratio, CI–Confidence interval

## Discussion

The prevalence of IPV among this population of pregnant women was found to be 11.2%. The prevalence of harassment, emotional/psychological abuse, severe combined abuse, and physical abuse from the CAS subscales were 13.0%, 12.3%, 8.9%, and 8.2%, respectively. The least prevalent form of violence was sexual violence which occurred in 3.3% of the participants. The factors found to be associated with IPV among this population of pregnant women were the employment status of the women, the experience of violence committed by parents or another family member in the past and current alcohol use by pregnant women and their partners.

The overall prevalence of IPV among pregnant women in this study was 11.2% which is lower than the global prevalence of 25.0% reported by Román-Gálvez et al in their systematic review and meta-analysis [2]. This prevalence is lower than those found in studies done in Asia (32.1%), North America (20.4%) and South America (25.6%) but higher than that of Europe (5.1%) [2] The prevalence of 11.2% in this study is slightly lower than the prevalence of 15% reported by Field et al in South Africa [16]. Differences in the sociocultural acceptance of violence against women and intimate partner violence (IPV) may be linked to the observed variations in the geographical distribution of IPV [2]. For instance, in many African social contexts such as Ghana, issues about marital conflict and abuse in marriage are hardly discussed, thus there is the possibility of under-reporting of IPV.

Studies within the African region have reported higher prevalence rates such as those by Adebowale et al Nigeria (24.8%) [28]; Fekadu et al and Azene et al (Ethiopia)–58.7% and 41.1% respectively; [7, 29] all of whose study settings were hospital-based within a secondary or tertiary facility. Since tertiary and secondary level facilities receive referrals from other facilities for further management, the variation and higher occurrence may be related to the differences in the antenatal clinic settings. Furthermore, cases of IPV may also be included in these referrals to secondary or tertiary facilities. It is known that the attitude of pregnant women towards violence has an impact on how accurately they record their experiences, hence the prevalence of IPV in this study may potentially be underestimated. Misreporting is possible due to ethical and social difficulties surrounding IPV and the sensitive nature of this topic [4].

Even though emotional/psychological violence in this study is the most common type of IPV, its prevalence (12.3%) is lower than the prevalence of emotional violence (18.7%) reported by Román-Gálvez et al. [2]. Generally, IPV rates were highest in Africa, except for psychological IPV which was greater in North America [2]. The prevalence rate of psychological IPV in pregnancy in Africa was reported to be 25.2% [2]. In Ghana, Otu-Nyarko et al reported a prevalence of emotional abuse among cases in a case-control study to be 88.9% which is higher than that reported in this study [19]. Emotional/psychological abuse was the most common type of abuse reported by Jatta et al in Gambia [30]. It can therefore be suggested that, within the African context, emotional/psychological violence is among the most common types of IPV. Community norms towards IPV, the acceptance of violence as a way of life by some women, lower female empowerment and polygamy could all contribute to this trend of psychological violence in these African countries [11, 31].

The participants’ employment status was one of the factors associated with IPV in this study. Being employed [AOR = 0.16 (95% CI: 0.05–0.47), p = 0.001] was protective from experiencing IPV. Out of the 269 participants, 76.9% were employed, which was less than their partners’ employment rates of 97.4%. For these pregnant women in the Ledzokuku Municipality, although the majority of the participants were in employment, efforts to promote gender equality through economic empowerment projects are still necessary. The findings of this current study corroborate the study of Field et al in South Africa who reported a positive association between IPV and the employment status of women, with pregnant women more likely to experience IPV if they were unemployed [16]. Men who commit violent acts take advantage of the fact that many African women are economically vulnerable and depend on their spouses for financial support and access to prenatal care [16, 32].

Several studies have reported a positive association between the alcohol-consuming habit of partners of pregnant women and the experience of IPV [5, 16, 19, 28, 29]. These findings corroborate the current study where pregnant women whose partners used alcohol had 10 times the odds of experiencing IPV compared to women whose partners didn’t use alcohol. Findings from this study support the systematic review and meta-analysis by Alebel et al. [33] and Bifftu and Guracho [34] in which pregnant women whose partners used alcohol were 11.4 times and 2 times, respectively, more likely to be abused when compared to their counterparts.

The strong association between alcohol use by partners and IPV could be because alcohol intake has a direct impact on consumers’ cognitive and physical functions. This mental distortion may result in aggressive behaviours in the relationship and a rise in violent incidents [29, 35]. Alcohol use may also heighten a partner’s sense of power and authority, enabling him or her to exert control over women [29]. From this current study, the alcohol-drinking habit of pregnant women was also a significant predictive factor of IPV and 5.6% of participants reported drinking alcohol. Contrary to these findings, Chasweka et al in Malawi found no association between alcohol consumption by pregnant women and IPV [36] in a study that employed a cross-sectional quantitative method among pregnant women attending an antenatal clinic in a District Hospital. The reason for no reported association between alcohol consumption by pregnant women and IPV could be due to the very low alcohol consumption rate of 0.7% among the study participants [36].

Studies have reported a statistically significant positive association between previous IPV exposure and IPV exposure during pregnancy [37, 38]. Among the victim-related factors associated with IPV from a systematic review and meta-analysis were the history of childhood violence and a family history of violence [34]. Pregnant women who had a history of childhood violence and a family history of violence were 3.14 times and 1.68 times respectively, more likely to experience IPV, [34] which was comparable to the findings of this current study. Luhumyo et al in Kenya [38] and Clarke et al in Uganda [37] found a significant association between IPV and a previous experience of IPV. IPV was more likely to occur during pregnancy in women who had previously experienced it [37]. Luhumyo et al reported a significant association between IPV and a previous experience of IPV such that having previously experienced IPV was linked to a 17 times greater risk of physical/sexual IPV compared to not having previously experienced IPV [38]. This was also a facility-based cross-sectional study but within a teaching and referral hospital. The social learning theory of IPV states that prior exposure to IPV is a background component that plays a role in the development and maintenance of aggression, which in turn causes IPV to occur during pregnancy [38].

### Limitations and strengths of the study

This study has contributed to the body of knowledge on IPV in pregnant women by highlighting the magnitude, pattern, and associated factors of IPV among pregnant women in a primary care setting in Ghana. The study utilized a standardized validated questionnaire with many items to measure different types of IPV. However, the cross-sectional nature of the study limits the ability to draw causal inferences from the associations established. Furthermore, the tool used to assess IPV required participants to answer questions about their intimate relationships over a 12-month period which has an inherent risk of recall bias/information bias.

## Conclusion

The findings of this study have illustrated a high prevalence (11.2%) of IPV among pregnant women attending a primary antenatal care clinic in a resource-limited setting. Healthcare providers in primary care such as family physicians, obstetricians, midwives, medical officers, and community health nurses need to recognize IPV as a medical condition that can occur in pregnancy and be ready to assist and manage the victims when it is detected. There is a need for policies by health service managers and opportunities to train healthcare workers in primary care to identify IPV. The implications of the association between the significant factors (ie. employment status, alcohol use by women or partners and previous history of violence) and IPV in clinical practice are that healthcare providers must be aware of the true impact of IPV to aid the development of successful IPV preventive strategies through screening during pregnancy. The findings from this study suggest that, in order to facilitate timely interventions, primary care antenatal clinics should incorporate targeted screening for IPV among pregnant women to provide opportunities to address this issue in primary care settings.

## Supporting information

S1 Table Chi-square test of associations. (DOCX)

S1 File Composite abuse scale dataset. (XLSX)

S2 File Questionnaire. (DOCX)
