# Supplementary material for: Intimate partner violence among pregnant women attending a low-resource primary care facility in Ghana
Source: PLoS One. 2024 Sep 9;19(9):e0310169. doi: 10.1371/journal.pone.0310169 (PMC11383221; doi:10.1371/journal.pone.0310169)
Supplement: S1 Table — (DOCX) [file pone.0310169.s001.docx]

| Association Between Socio-demographic Factors of Participants and Intimate Partner Violence | | | | |
| --- | --- | --- | --- | --- |
| Characteristics | Not abused  n ( % ) | Abused  n ( % ) | χ2 | p-value |
| Age group of participants (years) |  |  | 2.656 | 0.265 |
| 15 - 24 | 42 (85.7) | 7 (14.3) |  |  |
| 25 - 34 | 154 (88.0) | 21 (12.0) |  |  |
| 35 - 44 | 42 (95.6) | 2 (4.4) |  |  |
| Marital Status |  |  | 3.431 | 0.180 |
| Single | 47 (82.5) | 10 (17.5) |  |  |
| Married | 161 (89.9) | 18 (10.1) |  |  |
| Co-habitation | 31 (93.9) | 2 (6.1) |  |  |
| Length of Relationship (in years) |  |  | 4.103 | 0.251 |
| Less than 1 year | 28 (80) | 7 (20) |  |  |
| 1 - 5 | 158 (91.3) | 15 (8.7) |  |  |
| 6 - 11 | 41 (87.2) | 6 (12.8) |  |  |
| 11 years and above | 12 (85.7) | 2 (14.3) |  |  |
| Type of Marriage |  |  | 1.307 | 0.253 |
| Monogamous | 161 (91.0) | 16 (9.0) |  |  |
| Polygamous | 8 (80) | 2 (20) |  |  |
| Highest level of Education of Participants | |  | 2.347 | 0.504 |
| No formal education | 6 (100) | 0 (0.0) |  |  |
| Primary | 29 (82.9) | 6 (17.1) |  |  |
| Secondary | 152 (88.9) | 19 (11.1) |  |  |
| Tertiary | 52 (91.2) | 5 (8.8) |  |  |
| Employment Status of Participants | |  | 36.221 | 0.000* |
| Unemployed | 42 (67.7) | 20 (32.3) |  |  |
| Employed | 197 (95.2) | 10 (4.8) |  |  |
| Religion of participants |  |  | 0.2297 | 0.891 |
| Islam | 11 (91.7) | 1 (8.3) |  |  |
| Christianity | 227 (88.7) | 29 (11.3) |  |  |
| Traditional | 1 (100) | 0 (0.0) |  |  |

| Continuation  Association Between Socio-demographic Factors of Partners and Intimate Partner Violence | | | | |
| --- | --- | --- | --- | --- |
| Characteristics | Not abused  n ( % ) | Abused  n ( % ) | χ2 | p-value |
| Age group of partners (in years) |  |  | 4.973 | 0.174 |
| 15 - 24 | 8 (88.9) | 1 (11.1) |  |  |
| 25 - 34 | 109 (84.5) | 20 (15.5) |  |  |
| 35 - 44 | 103 (92.8) | 8 (7.2) |  |  |
| 45 - 54 | 19 (95.0) | 1 (5.0) |  |  |
| Partner’s employment status |  |  | 7.291 | 0.007* |
| Unemployed | 4 (57.1) | 3 (42.9) |  |  |
| Employed | 235 (89.7) | 27 (10.3) |  |  |
| Partner’s highest level of education |  |  | 13.038 | 0.005* |
| No formal education | 4 (57.1) | 3 (42.9) |  |  |
| Primary | 14 (73.7) | 5 (26.3) |  |  |
| Secondary | 133 (89.9) | 15 (10.1) |  |  |
| Tertiary | 88 (92.6) | 7 (7.4) |  |  |
| Partner’s religion |  |  | 4.149 | 0.246 |
| Islam | 9 (81.8) | 2 (18.2) |  |  |
| Christianity | 225 (89.6) | 26(10.4) |  |  |
| Traditional | 1 (50.0) | 1 (50.0) |  |  |
| Others | 4 (80.0) | 1 (20.0) |  |  |

(* statistically significant)

Association between Obstetric Factors and Intimate Partner Violence

| Characteristics | Not abused  n ( % ) | Abused  n ( % ) | χ2 | p-value |
| --- | --- | --- | --- | --- |
| Gravidity |  |  | 0.0041 | 0.949 |
| Primigravida | 49 (89.1) | 6 (10.9) |  |  |
| Multigravida | 190 (88.8) | 24 (11.2) |  |  |
| Parity |  |  | 3.984 | 0.263 |
| Nulliparity | 70 (87.5) | 10 (12.5) |  |  |
| Primiparity | 88 (93.6) | 6 (6.4) |  |  |
| Multiparity | 79 (84.9) | 14 (15.1) |  |  |
| Grand multiparity | 2 (100) | 0 (0.0) |  |  |
| Current Pregnancy Unplanned |  |  | 63.901 | 0.000* |
| Yes | 63 (67.7) | 30 (32.3) |  |  |
| No | 176 (100) | 0 (0.0) |  |  |
| Gestational age of Pregnancy |  |  | 2.776 | 0.250 |
| First trimester (1 to 12 weeks) | 26 (92.9) | 2 (7.1) |  |  |
| Second trimester(13 to 27 weeks) | 113 (85.6) | 19 (14.4) |  |  |
| Third trimester (28weeks to delivery) | 100 (91.7) | 9 (8.3) |  |  |
| Gestational age at booking |  |  | 11.183 | 0.004* |
| First trimester (1 to 12 weeks) | 123 (94.6) | 7 (5.4) |  |  |
| Second trimester(13 to 27 weeks) | 110 (84.6) | 20 (15.4) |  |  |
| Third trimester (28weeks to delivery) | 6 (66.7) | 3 (33.3) |  |  |
| History of miscarriage or abortion |  |  | 1.479 | 0.224 |
| Yes | 77 (85.6) | 13 (14.4) |  |  |
| No | 162 (90.5) | 17 (9.5 ) |  |  |
| History of stillbirth |  |  | 3.859 | 0.049* |
| Yes | 11 (73.3) | 4 (26.7) |  |  |
| No | 228 (89.8) | 26 (10.2) |  |  |
| History of preterm delivery |  |  | 3.047 | 0.081 |
| Yes | 4 (66.7) | 2 (33.3) |  |  |
| No | 235 (89.4) | 28 (10.6) |  |  |

(* statistically significant)

| Association Between Behavioural Factors and Intimate Partner Violence | | | | | |
| --- | --- | --- | --- | --- | --- |
| Characteristics | Not abused  n ( % ) | | Abused  n ( % ) | χ2 | p-value |
| Participant’s experience of conflict or dissatisfaction in the relationship |  | |  | 154.799 | 0.000* |
| Yes | 14 (33.3) | | 28 (66.7) |  |  |
| No | 225 (99.1) | | 2 (0.9) |  |  |
| Participant’s past experience of violence committed by parents or another family member |  | |  | 26.524 | 0.000* |
| Yes | 13 (56.5) | | 10 (43.5) |  |  |
| No | 226 (91.9) | | 20 (8.1) |  |  |
| Participant’s alcohol use in the last 12 months |  | |  | 38.254 | 0.000* |
| Yes | 6 (40.0) | | 9 (60.0) |  |  |
| No | | 233 (91.7) | 21 (8.3) |  |  |
| Partner alcohol use in the last 12 months |  | |  | 52.766 | 0.000* |
| Yes | 49 (66.2) | | 25 (33.8) |  |  |
| No | 190 (97.4) | | 5 (2.6) |  |  |
| Partner cigarette smoking in the last 12 months |  | |  | 1.705 | 0.260 |
| Yes | 3 (75.0) | | 1 (25.0) |  |  |
| No | 238 (89.8) | | 27 (10.2) |  |  |
| Partner use of recreational drugs in the last 12 months |  | |  | 1.705 | 0.260 |
| Yes | 3 (75.0) | | 1 (25.0) |  |  |
| No | 238 (89.8) | | 27 (10.2) |  |  |
| Participant PHQ 2 Screening for Depression |  | |  | 106.217 | 0.000* |
| Not Depressed | 231 (95.5) | | 11 (4.5) |  |  |
| Depressed | 8 (29.6) | | 19 (70.4) |  |  |
| (* statistically significant) | | | | | |
