# Supplementary material for: Intimate partner violence among pregnant women attending a low-resource primary care facility in Ghana
Source: PLoS One. 2024 Sep 9;19(9):e0310169. doi: 10.1371/journal.pone.0310169 (PMC11383221; doi:10.1371/journal.pone.0310169)

# **QUESTIONNAIRE**

1. **SOCIO-DEMOGRAPHIC DATA (**Please TICK (√) as appropriate)

Age: …………………… (Years)

Marital status: Single□ Married□ Separated□ Co-habitation□ Divorced□

Length of Relationship or Marriage (if married): ………………………… (Years)

Type of Marriage (if married): Monogamous□ Polygamous□

Employment status: Employed □ Unemployed □

Highest level of education: None at all□ Primary□ Secondary□ Tertiary□

Religion: Islam□ Christianity□ Traditional□ Other (specify)………………

**Partner Socio-demographic data**

Partner’s Age: …………………… (Years)

Partner’s Employment status: Employed □ Unemployed □

Partner’s Highest level of education: None at all□ Primary□ Secondary□ Tertiary□

Partner’s Religion: Islam□ Christianity□ Traditional□ Other (specify)………………

1. **OBSTETRIC CHARACTERISTICS**

Number of Pregnancies (Gravidity): 1□ 2□ 3□ 4□ 5 and above□

Number of Deliveries (Parity): 1□ 2□ 3□ 4□ 5 and above□

Current Pregnancy unplanned: Yes□ No□

Gestational age of pregnancy: …………………… (Weeks)

Gestational age at Booking: …………………… (Weeks)

History of miscarriage or abortion: Yes□ No□

History of stillbirth: Yes□ No□

History of Preterm Delivery: Yes□ No□

1. **BEHAVIOURAL FACTORS**

Are you experiencing conflict or dissatisfaction in your relationship? Yes□ No□

Have you experienced violence committed by your parents or another family member in the past? Yes□ No□

Have you used alcohol in the last 12 months? Yes□ No□

Have you smoked cigarettes in the last 12 months? Yes□ No□

Have you used other recreational drugs like marijuana in the last 12 months? Yes□ No□

Has your partner used alcohol in the last 12 months? Yes□ No□

Has your Partner smoked cigarettes in the last 12 months? Yes□ No□

Has your partner used other recreational drugs like marijuana in the last 12 months? Yes□ No□

**Patient Health Questionnaire‐2** (PHQ‐2)

| **Over the last *2 weeks*, how often have you been bothered by any of the following problems?** | **Not at all** | **Several days** | **More than half the days** | **Nearly every day** |
| --- | --- | --- | --- | --- |
| 1. Little interest or pleasure in doing things | 0 | 1 | 2 | 3 |
| 2. Feeling down, depressed, or hopeless | 0 | 1 |  | 3 |

1. COMPOSITE ABUSE SCALE (CAS)- Please circle

This section asks about your experiences in adult intimate relationships. By adult intimate relationship, we mean a husband, partner or boy/girlfriend for longer than one month.

1. Are you currently in a relationship? Yes.......1
 No .......2 Go to question 3

2. Are you currently afraid of your partner? Yes .......1
 No .......2
3. Have you ever been afraid of any partner? Yes .......1
 No .......2
4. Please circle the number, which matches the frequency, over a 12-month period that it happened to you.


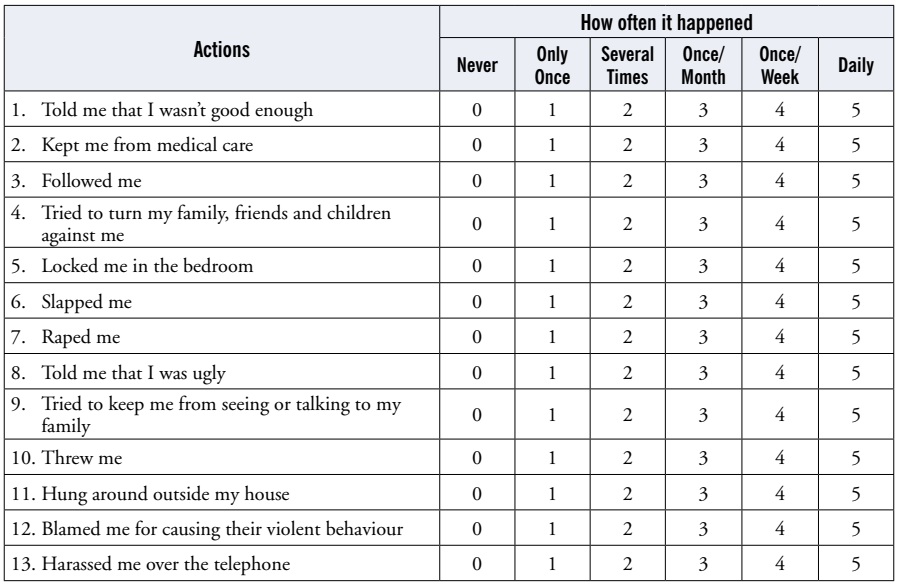


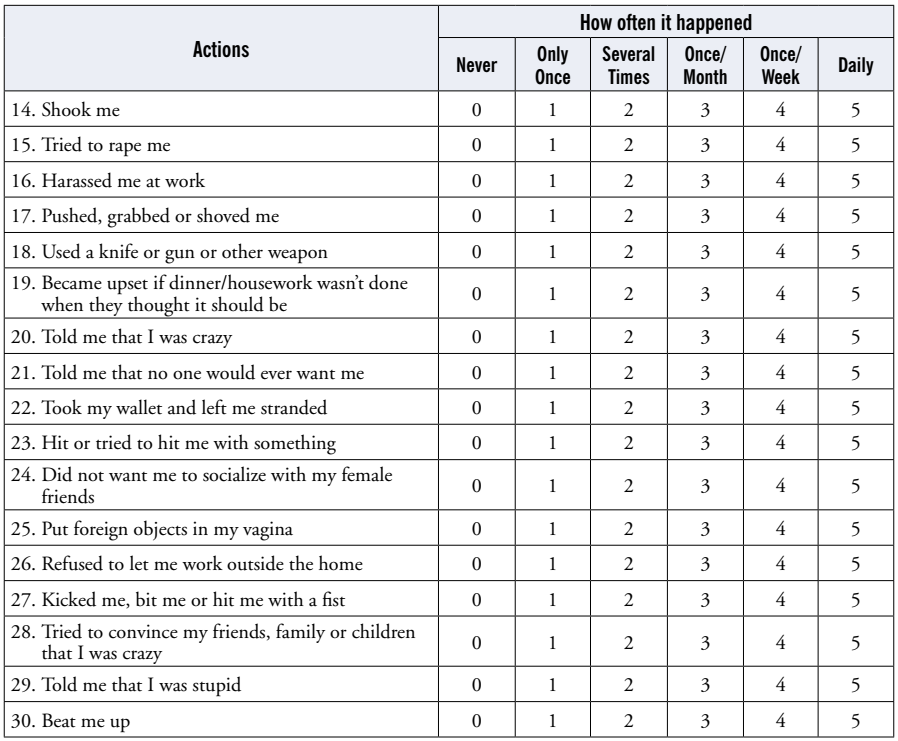

Supplement: S2 File — (DOCX) [file pone.0310169.s003.docx]
